# ABI3BP can inhibit the proliferation, invasion, and epithelial–mesenchymal transition of non-small-cell lung cancer cells

**DOI:** 10.1515/biol-2022-1034

**Published:** 2025-03-11

**Authors:** Jian Wu, Xiaokun Yan, Zewen Cheng

**Affiliations:** Uroth-thoracic surgery, Suzhou Hospital of Integrated Traditional Chinese and Western Medicine, No. 39 Xiashatang, Mudu Town, Wuzhong District, Suzhou, Jiangsu, 215100, China

**Keywords:** ABI3BP, non-small-cell lung cancer, MAPK/ERK pathway, epithelial–mesenchymal transition, tumor suppression

## Abstract

Lung cancer, especially non-small-cell lung cancer (NSCLC), has a poor 5-year survival rate below 20%, with factors like smoking, air pollution, and genetic mutations contributing to its development. ABI3BP, an extracellular matrix protein, inhibits NSCLC progression by regulating key signaling pathways; however, its exact mechanisms remain elusive. This study aimed to explore ABI3BP’s role in NSCLC and its impact on these pathways. We found that ABI3BP expression was significantly reduced in NSCLC cells compared to normal controls. Overexpression of ABI3BP in NSCLC cells resulted in a substantial reduction in cell growth and motility and induced cell cycle arrest. Furthermore, its overexpression suppressed the epithelial–mesenchymal transition (EMT) process in NSCLC cells. In addition, ABI3BP overexpression inhibited the MAPK/ERK pathway in NSCLC cells. Collectively, ABI3BP functions as a tumor suppressor in NSCLC by targeting the MAPK/ERK axis, thereby regulating cell proliferation, motility, and EMT. These findings suggest that ABI3BP represents a potential therapeutic target for NSCLC treatment.

## Graphical abstract

**Figure:**
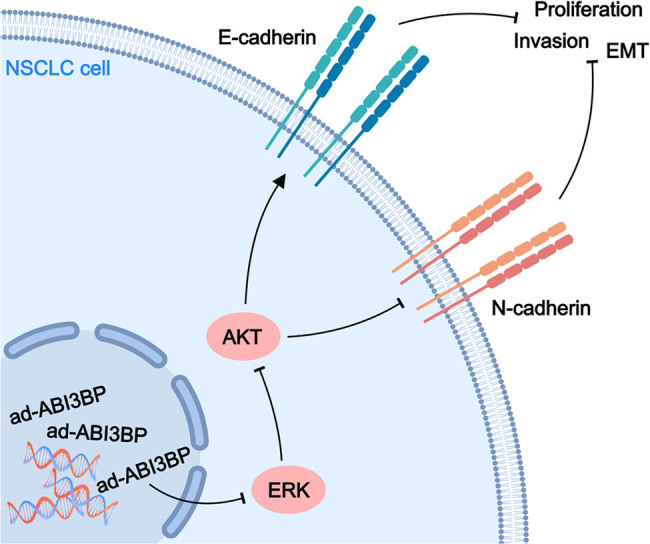

## 1 Introduction

Lung cancer remains one of the most prevalent malignancies worldwide, accounting for a significant proportion of cancer-related deaths in both men and women [1,2]. Non-small-cell lung cancer (NSCLC) represents approximately 85% of all lung cancer cases, with more than 60% of patients being diagnosed at advanced stages [3]. The 5-year survival rate for NSCLC remains dismal, at less than 20% [4]. Although smoking is a primary cause of lung cancer, a considerable number of cases occur in non-smokers due to factors such as air pollution, environmental exposure, genetic mutations, and single nucleotide polymorphisms [5]. These observations underscore the urgent need for novel diagnostic and therapeutic strategies that address both the etiology and progression of NSCLC.

The ABI family member 3 binding protein (ABI3BP) is a key extracellular matrix protein known to regulate cell proliferation, differentiation, and signal transduction in various tissues [6]. ABI3BP is integral to modulating key signaling pathways, particularly the MAPK/ERK pathway [7], which plays a vital role in cell proliferation, differentiation, and survival [8]. By inhibiting this pathway, ABI3BP can suppress uncontrolled cell growth, thereby impeding the progression of malignancies. Additionally, ABI3BP interacts with other critical pathways, including the PI3K/Akt and TGF-β cascades, both of which are involved in tumorigenesis and metastasis [6]. Through these interactions, ABI3BP exerts a wide range of biological effects, including cell cycle arrest, epithelial–mesenchymal transition (EMT) inhibition, and reduced invasive potential [6,9]. ABI3BP’s activity is not limited to lung cancer; it has also demonstrated tumor-suppressive functions in other malignancies, such as esophageal and breast cancers, where it downregulates oncogenic signals, making it a versatile tumor suppressor across different cancer types [10,11]. Notably, ABI3BP interacts with oncogenic pathways, potentially serving as a multifaceted regulator of tumor biology [12]. Reduced expression of ABI3BP has been associated with tumor progression and metastasis, highlighting its pivotal role in tumor suppression.

In NSCLC, however, the specific role of ABI3BP remains inadequately understood. This study investigates the therapeutic potential of ABI3BP in NSCLC, hypothesizing that it suppresses NSCLC progression through the regulation of key signaling pathways.

## 2 Materials and methods

### 2.1 Cell lines and cell culture

Human NSCLC cell lines HCC-827, A549, NCI-H460, and H1975, as well as normal bronchial epithelial cells (BEAS-2B), were purchased from ATCC. All cell lines were cultured in RPMI-1640 medium (Gibco, USA) supplemented with 10% fetal bovine serum (FBS, Gibco), 100 U/mL penicillin, and 100 μg/mL streptomycin (Gibco). Cells were maintained at 37°C in a humidified incubator with 5% CO_2_.

### 2.2 Reagents and antibodies

Adenoviral vectors expressing ABI3BP (ad-ABI3BP) and control adenovirus (ad-NC) were purchased from Vigene Biosciences (China). Western blot analyses were performed using primary antibodies from Abcam, including anti-ABI3BP (ab154189, 1:1,000), anti-E-cadherin (ab40772, 1:1,000), anti-N-cadherin (ab18203, 1:1,000), anti-α-SMA (ab5694, 1:1,000), anti-AKT (ab8805, 1:1,000), anti-phospho-AKT (ab38449, 1:1000), anti-ERK1/2 (ab184699, 1:1,000), and anti-phospho-ERK1/2 (ab201015, 1:1,000). Horseradish peroxide (HRP)-conjugated secondary antibodies were diluted at 1:5,000 (Abcam).

### 2.3 Adenovirus infection

Cells were infected with ad-ABI3BP or ad-NC adenoviruses at a multiplicity of infection of 100. The infection process was carried out for 24 h in serum-free medium, followed by incubation in complete medium for further experiments.

### 2.4 Immunoblot analysis

Samples were separated on sodium dodecyl sulfate-polyacrylamide gel electrophoresis gels and transferred to polyvinylidene difluoride membranes (Millipore, USA). After blocking with 5% non-fat milk, membranes were incubated overnight at 4°C with the primary antibodies, followed by incubation with HRP-conjugated secondary antibodies for 1 h at room temperature. Protein bands were visualized using an enhanced chemiluminescence detection kit (Beyotime), and band intensities were quantified using ImageJ software.

### 2.5 Cell viability assay

Cell viability was assessed using the Cell Counting Kit-8 (CCK-8, Beyotime) following the manufacturer’s instructions. Absorbance was measured at 450 nm using a microplate reader (Thermo Fisher, USA).

### 2.6 Migration and invasion assays

Cell migration and invasion were evaluated using Transwell assays (Corning, USA). For the migration assay, 1 × 10⁵ cells in serum-free medium were placed in the upper chamber of a Transwell insert. The lower chamber was filled with RPMI-1640 medium containing 10% FBS. For the invasion assay, the upper chamber was pre-coated with Matrigel (Corning), and 2 × 10⁵ cells were seeded. After 24 h, cells on the lower surface of the insert were fixed in 4% paraformaldehyde and stained with 0.1% crystal violet. The number of migrated or invaded cells was counted in five randomly selected fields using a light microscope (Zeiss, Germany).

### 2.7 FCM assay

The cells were washed with PBS and fixed using 70% ethanol at −20°C for 2 h. Subsequently, the cells were stained with propidium iodide at 4°C. The stained cells were then analyzed using a flow cytometer (BD, USA).

### 2.8 Statistical analysis

Data are expressed as mean ± standard deviation (SD). Statistical significance was determined using one-way ANOVA followed by Tukey’s post hoc test for multiple comparisons. A *p*-value of <0.05 was considered statistically significant.

## 3 Results

### 3.1 ABI3BP expression was significantly downregulated in NSCLC cells

**Figure 1:**
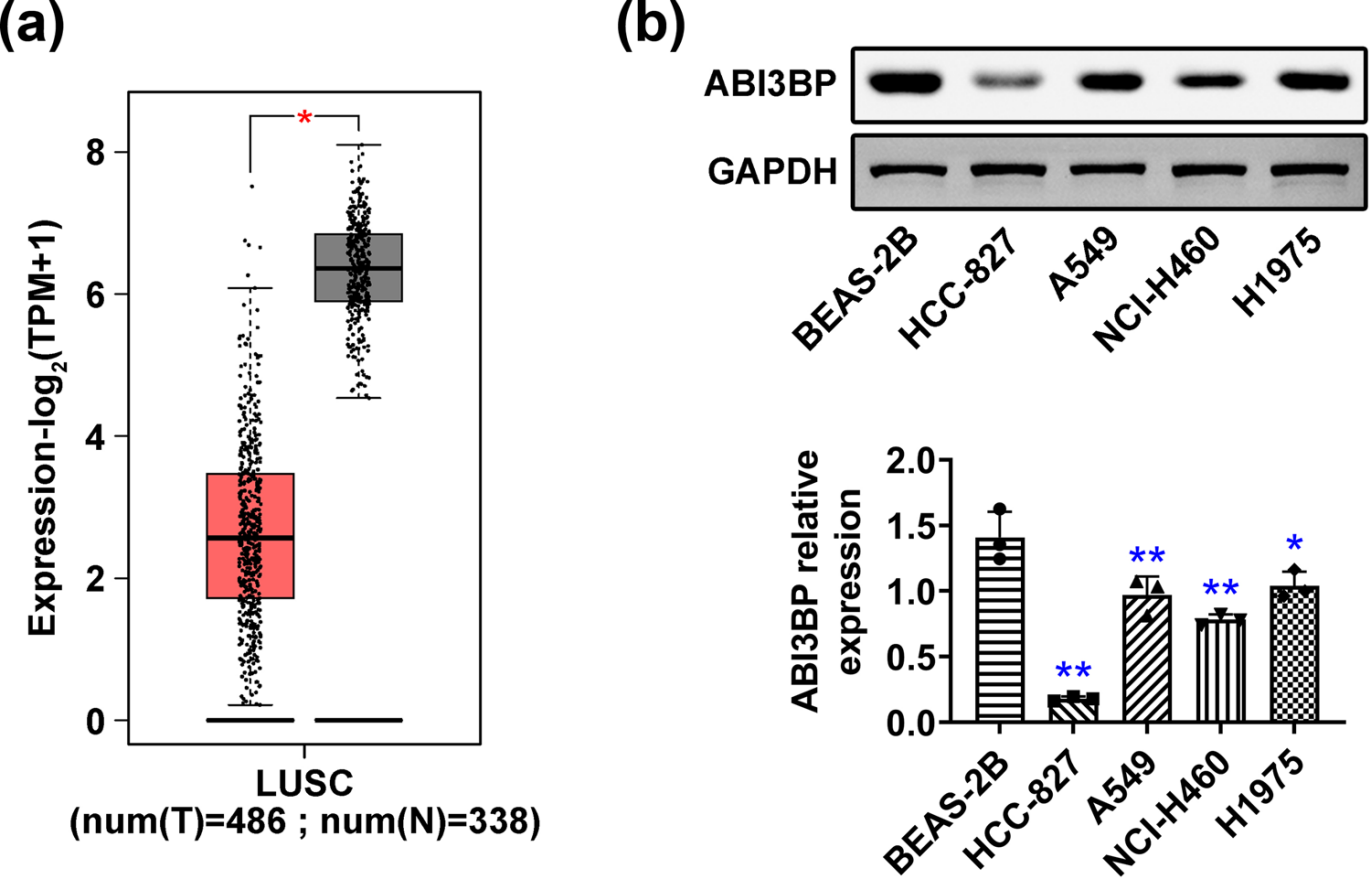
ABI3BP expression was significantly downregulated in NSCLC cells. (a) TCGA database showed the TPM (transcripts per million) value of ABI3BP in 338 normal tissues and 486 tumor tissues. (b) Immunoblot showing ABI3BP protein expression levels in normal bronchial epithelial cells (BEAS-2B) and NSCLC cell lines (HCC-827, A549, NCI-H460, H1975). Quantification of ABI3BP protein expression was shown. Data are represented as mean ± SD. *p < 0.05, **p < 0.01. NSCLC, non-small-cell lung cancer.

To assess the expression of ABI3BP in NSCLC, we first measured its levels through the TCGA database. We observed that ABI3BP had a lower TPM value in NSCLC tissues compared to normal tissues (Figure 1a). Subsequently, we detected the expression of ABI3BP in both normal bronchial epithelial cells (BEAS-2B) and NSCLC cell lines (HCC-827, A549, NCI-H460, and H1975). Immunoblot analysis revealed that ABI3BP expression was significantly lower in all NSCLC cell lines compared to normal bronchial epithelial cells (Figure 1b). Quantification of ABI3BP protein levels confirmed the marked reduction in its expression in NSCLC cells (Figure 1b). Thus, ABI3BP expression was significantly downregulated in NSCLC cells.

### 3.2 Overexpression of ABI3BP in NSCLC cells led to a marked reduction in cell growth as well as motility

**Figure 2:**
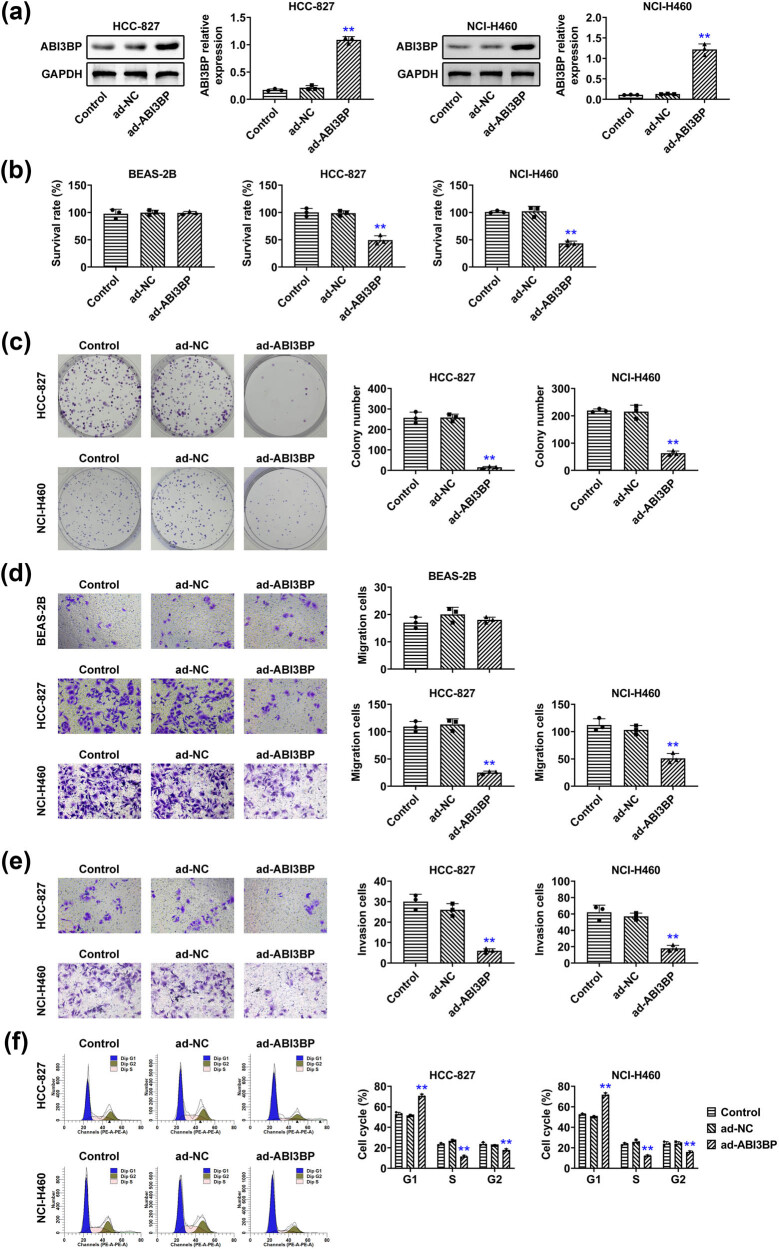
Overexpression of ABI3BP in NSCLC cells led to a marked reduction in cell growth as well as motility. (a) Immunoblot showing ABI3BP protein levels in HCC-827 and NCI-H460 cells upon the indicated infection. GAPDH was used as the loading control. Quantification of relative ABI3BP expression is shown on the right. (b) Cell viability assay (CCK8) results for BEAS-2B, HCC-827, and NCI-H460 cells following overexpression of ABI3BP. The survival rate (%) is shown for the control, ad-NC, and ad-ABI3BP groups. (c) Colony formation assays showed the effects of ABI3BP overexpression on the proliferation of HCC-827 and NCI-H460 cells. The colony numbers were counted. (d) Transwell migration assay of BEAS-2B, HCC-827, and NCI-H460 cells with images showing migrated cells in each group. Quantification of migrated cells is shown on the right. (e) Transwell invasion assay of HCC-827 and NCI-H460 cells with images showing invaded cells in each group. (f) FCM assays showed the effects on the cell cycle of HCC-827 and NCI-H460 cells. The cells at different phases were calculated. Quantification of invaded cells is shown on the right. Data are represented as mean ± SD. **p < 0.01, ad-ABI3BP vs ad-NC. NSCLC, non-small-cell lung cancer. NC, negative control.

Next, we evaluated the effect of ABI3BP overexpression on cell viability using the CCK8 assay. HCC-827 and NCI-H460 cells were transfected with either control (ad-NC) or ABI3BP-expressing adenovirus (ad-ABI3BP), and the overexpression efficiency was confirmed by Immunoblot (Figure 2a). Further, CCK-8 assays showed that overexpression of ABI3BP led to a significant decrease in cell viability in both NSCLC cell lines compared to the control and ad-NC groups (Figure 2b). However, its overexpression has modest effects on the viability of BEAS-2B cells (Figure 2b). Colony formation assays further confirmed that the overexpression of ABI3BP suppressed the proliferation of HCC-827 and NCI-H460 cells (Figure 2c). Transwell migration and invasion assays were performed using HCC-827 and NCI-H460 cells. The results indicated that overexpression of ABI3BP significantly reduced the migratory capacity of both cell lines compared to the control groups (Figure 2d). In contrast, ABI3BP overexpression did not affect the migration of BEAS-2B cells, as confirmed by Transwell assays (Figure 2d). Additionally, the invasive ability of HCC-827 and NCI-H460 cells was also markedly suppressed following ABI3BP overexpression (Figure 2e). Further, the overexpression of ABI3BP led to the cell cycle arrest in HCC-827 and NCI-H460 cells (Figure 2f). Therefore, overexpression of ABI3BP in NSCLC cells resulted in a significant reduction in cell growth as well as motility.

### 3.3 ABI3BP overexpression suppressed the EMT process of NSCLC cells

**Figure 3:**
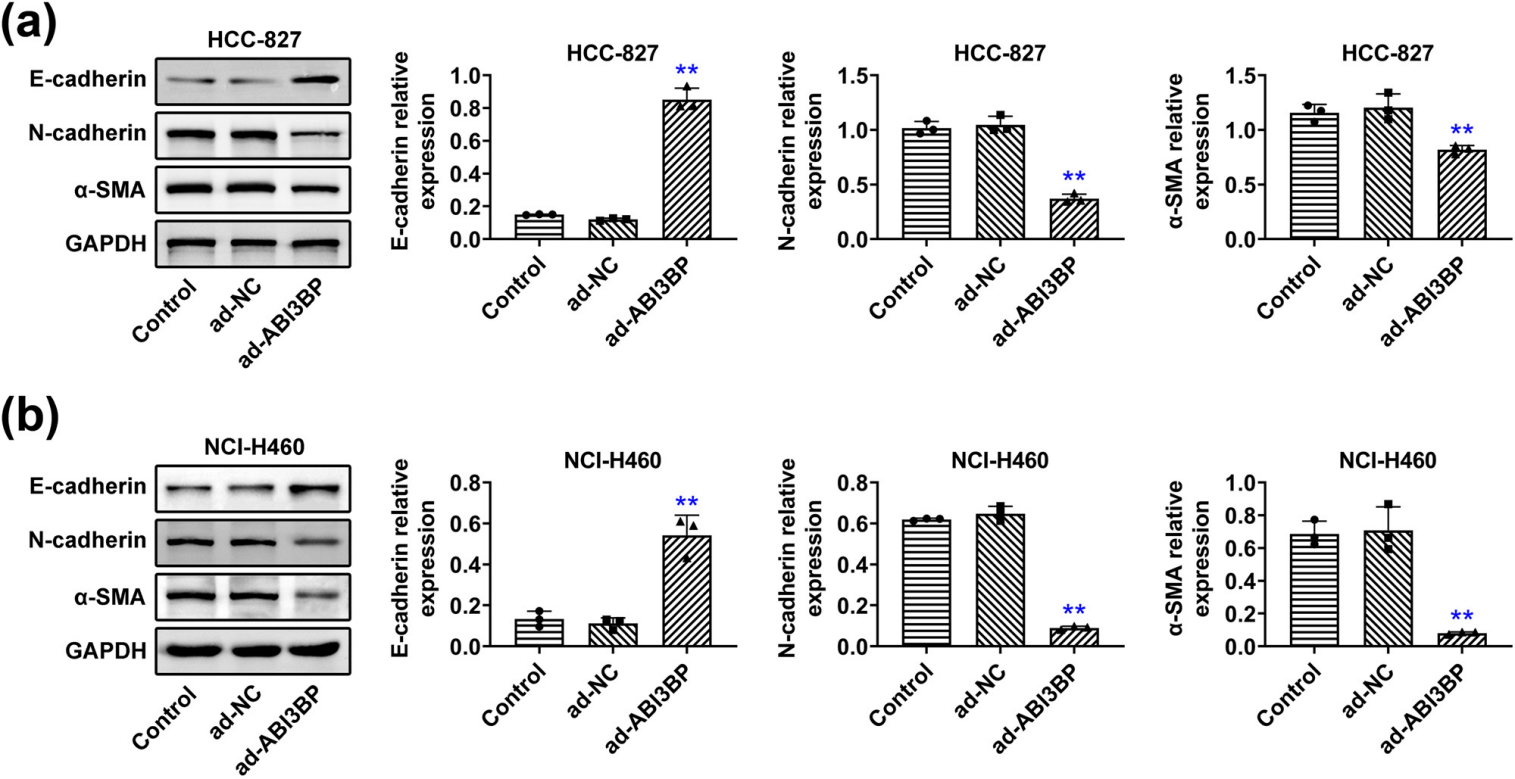
ABI3BP overexpression suppressed the EMT process of NSCLC cells. (a) and (b) Immunoblot showing E-cadherin, N-cadherin, and α-SMA protein levels in HCC-827 (a) and NCI-H460 (b) cells upon the indicated infection. GAPDH was used as the loading control. Quantification of relative E-cadherin, N-cadherin, and α-SMA expression is shown on the right. Data are represented as mean ± SD. **p < 0.01, ad-ABI3BP vs ad-NC. NSCLC, non-small-cell lung cancer. NC, negative control.

Since EMT plays a crucial role in cancer metastasis, we examined whether ABI3BP influences EMT markers in NSCLC cells. Western Immunoblot analysis revealed that ABI3BP overexpression increased the expression of the epithelial marker E-cadherin and decreased the expression of mesenchymal markers N-cadherin and α-SMA in both HCC-827 (Figure 3a) and NCI-H460 (Figure 3b) cells. These findings suggest that ABI3BP inhibits EMT, thereby reducing the invasive potential of NSCLC cells.

### 3.4 ABI3BP overexpression suppressed MAPK/ERK pathway in NSCLC cells

**Figure 4:**
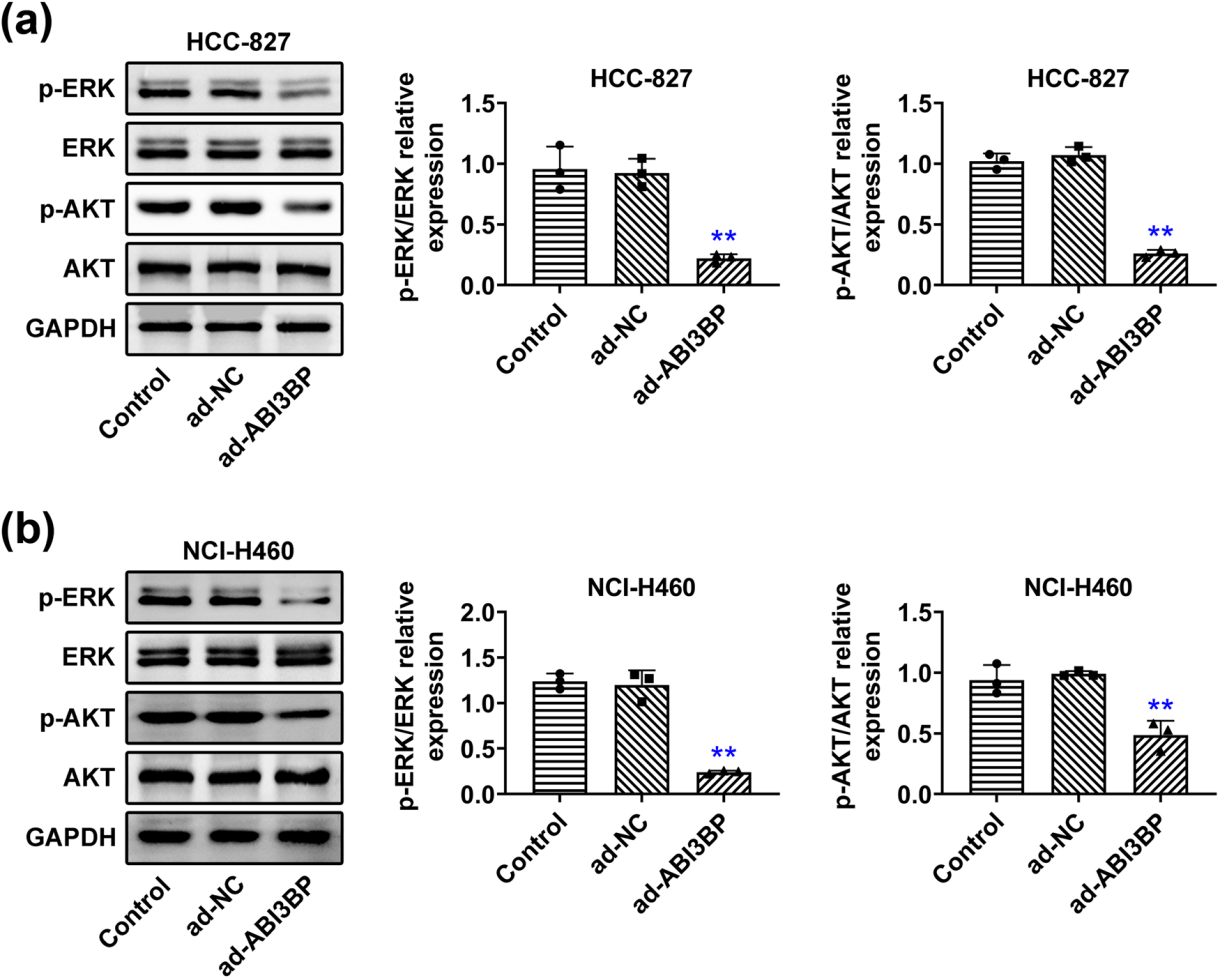
ABI3BP overexpression suppressed the MAPK/ERK pathway in NSCLC cells. (a) and (b) Immunoblot showing ERK and AKT protein levels and phosphorylation levels in HCC-827 (a) and NCI-H460 (b) cells upon the indicated infection. GAPDH was used as the loading control. Quantification of relative phosphorylation levels of ERK and AKT is shown on the right. Data are represented as mean ± SD. **p < 0.01, ad-ABI3BP vs ad-NC. NSCLC, non-small-cell lung cancer. NC, negative control.

To explore the molecular mechanisms by which ABI3BP regulates NSCLC cell proliferation and migration, we investigated the MAPK/ERK pathway. Immunoblot analysis revealed that overexpression of ABI3BP reduced the phosphorylation of ERK and Akt in both HCC-827 (Figure 4a) and NCI-H460 (Figure 4b) cells, indicating that ABI3BP inhibits the MAPK/ERK pathway. These results suggest that ABI3BP suppresses NSCLC progression by modulating the MAPK/ERK pathway.

## 4 Discussion

NSCLC is the most prevalent form of lung cancer, comprising around 85% of all lung cancer diagnoses. It is highly aggressive, characterized by rapid cell proliferation, migration, and EMT, which collectively drive tumor progression and metastasis [13,14,15,16]. These biological processes are regulated by several key signaling pathways and proteins that ensure proper cellular function. Disruption of these pathways often leads to poor clinical outcomes in NSCLC patients. In our study, we explored the role of ABI3BP in regulating cell proliferation, migration, and EMT, providing insights into its potential therapeutic relevance in NSCLC.

EMT plays a crucial role in advancing cancer by enhancing the metastatic ability of cancer cells [17]. Through this process, epithelial cells acquire mesenchymal traits, thereby increasing their capacity for migration and invasion. EMT is also implicated in resistance to cancer therapies, making it a critical target in NSCLC treatment strategies. Our findings revealed that ABI3BP overexpression led to a significant reduction in EMT in NSCLC cells, as evidenced by altered levels of EMT markers, such as E-cadherin and N-cadherin. These results underscore the importance of targeting EMT to limit the metastatic potential of NSCLC.

ABI3BP, a vital extracellular matrix protein, is involved in the regulation of various cellular functions such as cell growth, differentiation, and programmed cell death. Its role as a tumor suppressor has been established in multiple cancer types, including esophageal and gallbladder cancers, where it inhibits cellular proliferation and migration [10,11]. ABI3BP’s interactions with key signaling pathways, including MAPK/ERK and PI3K/Akt, are essential for its tumor-suppressive activities. In this study, we observed that overexpression of ABI3BP in NSCLC cells led to reduced proliferation and invasion, consistent with its known role as a tumor suppressor.

Beyond these general cellular effects, ABI3BP exerts specific control over EMT, as well as cell proliferation and migration in NSCLC [18]. Our research demonstrated that ABI3BP overexpression decreased EMT-related markers, notably reducing N-cadherin levels while increasing E-cadherin expression. In addition, ABI3BP was shown to suppress NSCLC cell migration and invasion, suggesting that its inhibition of these processes may be a crucial factor in reducing tumor progression and metastasis. This highlights ABI3BP as a significant modulator of EMT and an inhibitor of NSCLC progression.

ABI3BP overexpression inhibited the MAPK/ERK pathway in NSCLC cells. Previous studies have reported that the MAPK/ERK pathway plays critical in mediating cancer cell proliferation, and its alteration could affect the proliferation of cancer cells, including NSCLC cells [19,20]. The MAPK/ERK and PI3K/Akt pathways are pivotal in regulating cellular processes like proliferation, migration, and survival. Dysregulation of these pathways is common in cancer and is linked to enhanced tumor growth and metastasis [19,20]. The MAPK/ERK pathway is primarily activated in response to growth stimuli, facilitating cell proliferation and survival, while the PI3K/Akt pathway similarly drives cell growth and migration. Both pathways are intimately involved in regulating EMT, and their dysregulation accelerates NSCLC progression. In this study, we found that ABI3BP suppresses the activation of both ERK and Akt, thereby inhibiting NSCLC cell proliferation and EMT. These findings suggest that modulating these pathways through ABI3BP could be a promising approach to NSCLC therapy.

We propose that future research could include in vivo tumor formation experiments in nude mice to further validate the conclusions and clarify the downstream molecular mechanism through multi-omics analysis. Moreover, while we focused on specific pathways, other signaling networks may also contribute to the tumor-suppressive effects of ABI3BP that have not yet been explored. Future research should aim to expand on these findings and investigate the clinical applications of ABI3BP as a therapeutic target.

In summary, our study highlights the tumor-suppressive role of ABI3BP in NSCLC, revealing its ability to regulate key signaling pathways involved in cell proliferation, migration, and EMT. ABI3BP presents a promising target for therapeutic strategies aimed at controlling NSCLC progression. Further research into its mechanistic interactions could lead to the development of novel treatments for NSCLC patients, potentially improving their prognosis.
